# Optically Transparent Carbon Electrodes for Single Entity Electrochemistry

**DOI:** 10.1021/acselectrochem.4c00048

**Published:** 2024-10-08

**Authors:** Kelly L. Vernon, Tipsiri Pungsrisai, Oluwasegun J. Wahab, Sasha E. Alden, Yaxu Zhong, Myung-Hoon Choi, Ekta Verma, Anne K. Bentley, Kathleen O. Bailey, Sara E. Skrabalak, Xingchen Ye, Katherine A. Willets, Lane A. Baker

**Affiliations:** † Department of Chemistry, Texas A&M University, College Station, Texas 77843, United States; ‡ Department of Chemistry, Temple University, Philadelphia, Pennsylvania 19122, United States; § Department of Chemistry, Indiana University, Bloomington, Indiana 47405, United States; ∥ Department of Chemistry, Lewis & Clark College, Portland, Oregon 97219, United States

**Keywords:** Scanning electrochemical cell microscopy, single entity electrochemistry, electrodissolution, optoelectrochemistry, optically transparent carbon electrode, indium tin oxide electrode

## Abstract

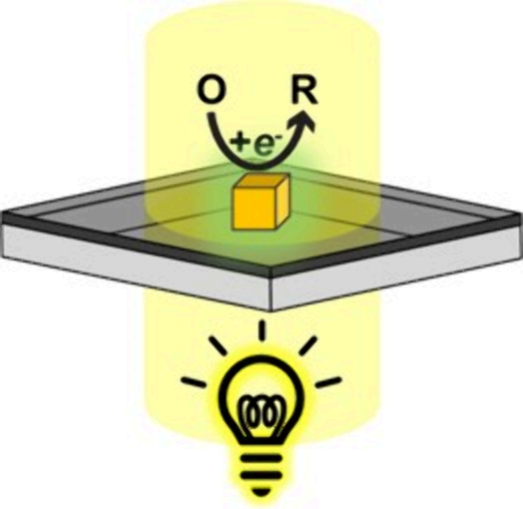

We demonstrate the application and benefit of optically transparent carbon electrodes (OTCEs) for single entity nanoelectrochemistry. OTCEs are prepared by pyrolyzing thin photoresist films on fused quartz coverslips to create conductive, transparent, thin films. Optical, electrical, topographical, and electrochemical properties of OTCEs are characterized to evaluate their suitability for single entity electrochemistry. Nanoscale electrochemical imaging of the OTCEs using scanning electrochemical cell microscopy (SECCM) revealed uniform electrochemical activity for reduction of the hexaammineruthenium(III) redox complex, that was comparable to Au-coated glass, and in contrast to the heterogeneity observed with commonly used indium tin oxide (ITO) substrates. Additionally, we demonstrate the utility of the prepared OTCEs for correlative SECCM—scanning electron microscopy studies of the hydrogen evolution reaction at the surface of Au nanocubes. Lastly, we demonstrate the benefit of OTCEs for optoelectrochemical experiments by optically monitoring the electrodissolution of Au nanocrystals. These results establish OTCE as a viable transparent support electrode for multimode electrochemical and optical microscopy of nanocrystals and other entities.

## 1 Introduction

An underlying goal of single-entity electrochemistry (SEE) is to enable the study of intrinsic activities of individual nanoparticles to allow direct correlation of nanoparticle properties (e.g., size, shape, surface structure, composition) to electrochemical properties.^1,2^ Correlative multimodal scanning electrochemical cell microscopy (SECCM) approaches have gained traction for the SEE studies of nanoparticles.^3^ These approaches employ multiple analytical characterization techniques to develop a more thorough understanding of the structure-function relationship of particles studied. Recent examples of nanoparticle studies with this approach include *operando* SECCM with optical microscopy^4−6^ and co-located SECCM with surface characterization techniques.^7−13^ Notably, correlative SEE approaches require a support electrode that enables translation among the various analytical modalities involved. Ideal support electrodes are electrically conductive yet electrocatalytically inert compared to the material of interest,^14^ with additional required properties dependant on the techniques being engaged for study. For multimodal SEE studies, optical transparency, low surface roughness, and homogeneous electrochemical properties prove especially important.

For nanoparticle studies that utilize optical techniques, the most widely used transparent support electrode is indium tin oxide (ITO). The favorable conductivity and transparency to UV and visible light^15,16^ make ITO a useful platform for photovoltaics,^17^ electrochromics,^18^ sensors,^19,20^ fuel cells,^21^ and electrocatalysis.^22^ ITO is also popularly used for (super-resolution) optical microscopy of electrochemical processes such as electrodeposition,^6,23−25^ electrodissolution,^26−28^ and photocatalysis.^29−31^ However, ITO support electrodes have been shown to deteriorate under acidic reducing environments,^32−34^ a concern for studies of reactions such as the hydrogen evolution reaction (HER).^35^ Evidence of nanoscale heterogeneity in the electrochemical properties of ITO has also emerged. Studies of model redox probes,^36^ localized electrochemical nucleation,^37,38^ and nanoparticle dissolution^39^ have raised concerns over the impact the underlying ITO electrode has on measurements at single nanoparticles. Despite these drawbacks, ITO continues to be used prominently in correlative multi-microscopy,^4,5,10,14,40−43^ largely due to the lack of a suitable replacement.

**Figure 1:**
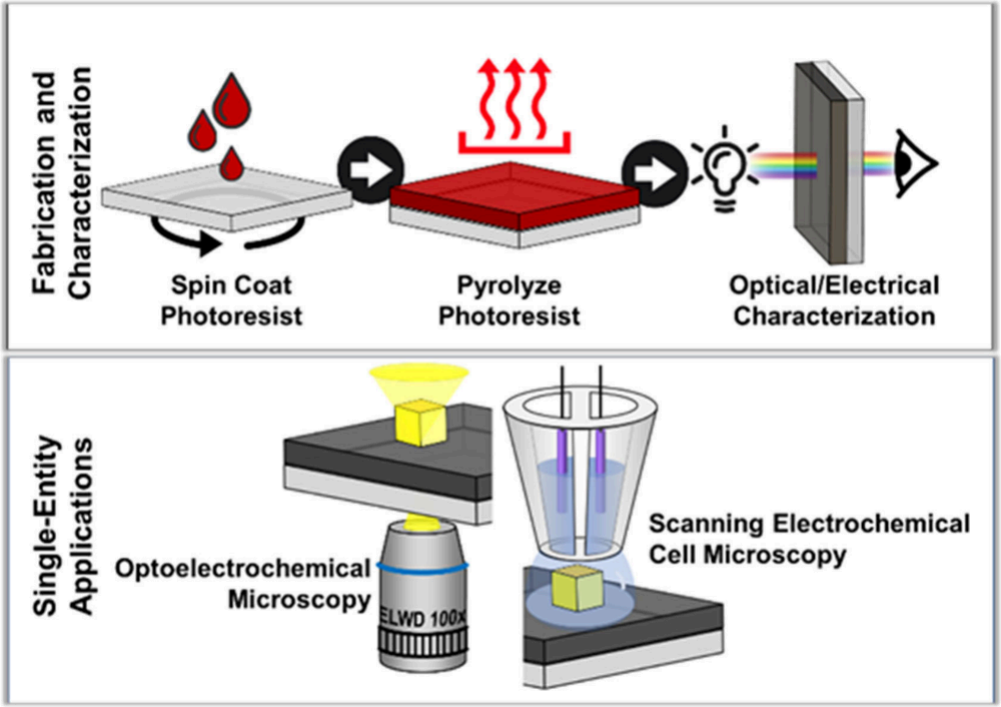
(Top panel) Schematic of OTCE fabrication process. (Bottom panel) Schematic of OTCEs in-use for SEE. Left: an OTCE used as a support electrode in an optoelectrochemical measurement using dark-field microscopy. Right: an OTCE used as a support electrode in scanning electrochemical cell microscopy.

Optically transparent carbon electrodes (OTCEs) present a possible surrogate for ITO electrodes, especially for correlative SEE (Figure 1). Carbon is a popular electrode material due to a wide operational window, low background current, low cost, and wide applicability to a broad range of redox systems.^44,45^ OTCEs can be fabricated in a straightforward fashion from pyrolyzed photoresist films (PPFs) (Figure 1, top). The macroscale electrochemical and optical properties of these PPF-derived carbon films have been studied previously^46−50^ and OTCES have been applied in a number of different venues.^47,50−52^ Specifically, Kim et al. investigated the electrochemical properties of PPFs, establishing their fundamental properties.^46^ Ranganathan et al. successfully utilized PPF electrodes in electrochemical applications and microelectromechanical systems.^47^ Building upon this work, Donner et al. demonstrated by adjusting film thickness, PPFs could be employed to fabricate OTCEs,^44^ and Stevenson’s group and others later expanded spectroelectrochemistry applications of OTCEs.^47,50−52^

In this report, we describe application of OTCEs as a homogeneous support electrode, detailing the technical merit of this electrode format for SEE. OTCEs are characterized via SECCM, atomic force microscopy (AFM), UV-visible spectroscopy, and conductivity measurements. Nanoscale electrochemical homogeneity of OTCEs was assessed via SECCM in comparison to ITO and Au films. We further demonstrate OTCEs as an appropriate support electrode for correlative SECCM-scanning electron microscopy (SEM) studies of HER as well as for optoelectrochemical monitoring of the dissolution of gold nanocubes (Au NCs). Lastly, we highlight the simplicity and generality of OTCEs through lab-to-lab reproducibility of OTCE fabrication. From these results, a compelling case for further application of OTCEs in SEE measurements is made.

## Experimental Section

### Chemicals

Hexaammineruthenium(III) chloride (Ru(NH_3_)_6_Cl_3_), potassium chloride (KCl), propylene glycol methyl ether acetate (PGMEA), sulfuric acid (H_2_SO_4_), perchloric acid (HClO_4_, 60% or 70%), potassium bromide (KBr, ≥ 99.0%), gold(III) chloride trihydrate (HAuCl_4_·3H_2_O, ≥99.9% trace metals basis), l-ascorbic acid (AA, ≥99.5%), silver nitrate (AgNO_3_, ≥99.0%), sodium borohydride (NaBH_4_, 99%), hydrochloric acid (HCl, 37 wt % in water), and nitric acid (HNO_3_, 70%), were used as received from Sigma-Aldrich. Hydrogen peroxide (H_2_O_2_, 30 wt %, Lab Alley), potassium perchlorate (KClO_4_, 99%, Alfa Aesar), hexadecyltrimethylammonium bromide (CTAB, >98.0%, TCI America), and potassium bromide (KBr, 99.999%, for Au NC synthesis, Acros Organics) were used as received. Aqueous solutions for Au NC electrodissolution studies were prepared with nanopure water (18.2 MΩ·cm, arium pro, Sartorius). Aqueous solutions for characterization and SECCM were prepared with Milli-Q water (18.2 MΩ·cm at 25 °C, Thermo Scientific). Ultrapure water (18.2 MΩ·cm at 25 °C) obtained from a Barnstead GenPure water purification system (Thermo Scientific) was used in nanoparticle synthesis experiments. All glassware for nanoparticle synthesis was cleaned with aqua regia (a mixture of HCl and HNO_3_ (v:v = 3:1)), rinsed thoroughly with water and dried before use.

### Materials

Fused quartz coverslips (1 × 1 in, 200 μm thick) were used as received from Technical Glass Products, Inc. Indium tin oxide (ITO) coated coverslips (22 × 26 nm, Thickness #1, 70-100 Ω resistivity) were used as received from Structure Probe, Inc. for SECCM measurements and solvent-cleaned and used in Au NC electrodissolution studies (see *Electrodissolution of Au NCs*) while another set of ITO coated coverslips from the same company (18 × 18 mm, Thickness #1, 8-12 Ω resistivity) were used for further SECCM measurements (SI, Section S8, Figure S12). Conductive silver epoxy (ETC-bond 556, Electron Microscopy Sciences), copper wire (300 V Solid Type 22 gauge, NTE Electronics), silver wire (1.0 mm diameter, 99.9% trace metals basis, Alfa Aesar) and platinum wire (1.0 mm diameter, 99.995% trace metals basis, Beantown Chemical) were used as received. Kapton tape was used as received from Thomas Scientific. SecureSeal Imaging Spacers (9 mm ID × 0.12 mm depth, 18 × 18 mm) were used as received from Grace Bio-Labs.

### Preparation of Optically Transparent Carbon Electrodes (OTCEs)

Fused quartz coverslips were first cleaned by soaking in piranha solution (**Caution**: *piranha is a strong oxidizing agent and can react violently with organic material*), for at least 30 min. Coverslips were then rinsed with DI water, dried under nitrogen, and baked on a hot plate at 250 °C for >2 h. Cleaned coverslips were allowed to cool for 20 seconds and then spin-coated (BIDTEC SP100 Spin Coater) with ∼0.2 mL of dilute S1813 photoresist (Microposit S1813, DOW), in 1:5 v/v dilution with PGMEA. A 2-step program was used for the spin-coating and consisted of a pre-spin (Time: 5 s, Speed: 500 rpm, Acceleration: 5), and a main spin (Time: 45 s, Speed: 3000 rpm, Acceleration: 5). The photoresist-coated coverslip was soft-baked on a hot plate at 110 °C for 5 mins. Pyrolysis of the soft-baked spin-coated coverslip in a tube furnace (MINIBRUTE Oxidation/Anneal Furnace) under a reducing atmosphere (5% H_2_, 95% Ar_2_ flowing at a rate of 1000 sccm) was carried out as follows: the temperature was ramped to 1000 °C at a rate of 20 °C/min, followed by holding the temperature at 1000 °C for 60 min, and finally ramping the temperature back to room temperature at 20 °C/min. The thickness of the resulting OTCEs was characterized by atomic force microscopy (AFM, XE-Bio system, Park Systems), and the sheet resistance (Four-Point Probe, Ossila), and transmittance (Cary 50 UV-visible Spectrophotometer, Varian) were also measured. Additional details of Au NC synthesis and macroscale electrochemical characterization are described in the Supporting Information.

### SECCM Measurements

Local electrochemical measurements were obtained with SECCM voltammetric mapping, operated as detailed in our previous work.^53^ Briefly, dual barrel nanopipettes were used as the scanning probe (SI, Section S1, Figure S1), with both barrels filled with electrolyte solution, and a quasi-reference counter electrode (QRCE) back-inserted into each barrel. A small potential is applied between the two electrodes to generate an ion current and the pipette dithers in the z axis. The AC component of the ion current serves to provide feedback as the tip approaches the surface.^54^ The meniscus formed between the nanopipette tip and the substrate creates an electrochemical cell, with the area of the substrate under the meniscus being the effective working electrode (WE). A cyclic voltammogram (CV) or linear sweep voltammogram (LSV) is then acquired, after which the nanopipette is retracted and moved to a new spot, affording spatially resolved voltammetric measurements that can be presented as electrochemical activity maps of the substrate.^54^ An environmental chamber was used to constantly purge the chamber with humidified argon gas. Additional details on the design have been detailed in a previous study.^53,55^ All potentials measured via SECCM are referenced versus 3.5 M KCl Ag/AgCl.

Sample preparation for HER measurements followed previous procedures described by Choi et al.^53^ Au NCs (see SI, Section S2 for synthesis) were dispersed across OTCE via electrospray (SI, Section S3). Additional removal of CTAB ligands from the sample was performed by submerging the sample in a MeOH bath for 2 min then drying with air, followed by submerging in Milli-Q water and again drying with air. Electrochemical cleaning was performed on the sample with a potentiostat (CH Instruments) from 0 to -1 V vs Ag/AgCl in 100 mM HClO_4_ for two cycles. A TEM index grid (Style 200F1, Cu, 3 mm, Structure Probe, Inc.) is used to identify areas with good distribution in SEM to be located on SECCM.

### Sample Preparation for Electrodissolution of Au NCs

ITO coverslips (70-100 Ω) were sonicated in acetone, isopropanol, then nanopure water for 20 min in each solvent. OTCE substrates were rinsed with nanopure water and dried with N_2_. The OTCE substrates were then air-plasma cleaned (PDC-32G, Harrick Plasma) for 10 s before use. A 2-inch-long Cu wire was attached to each substrate with conductive silver epoxy ETC-Bond 556 and heated until the epoxy was completely dry. Then, 75 μL of CTAB-capped Au NC solution (1:400 v/v dilution with nanopure water for ITO samples and 1:640 v/v with nanopure water for OTCE samples) was airbrushed (PointZero Dual-Action 7 cc Gravity-Feed airbrush, 0.3 mm nozzle, 5 psi N_2_) directly onto the center of the substrate^56^ and the sample was allowed to dry overnight in ambient conditions. As described previously,^57^ the presence of CTAB in Au NC-deposited samples was reduced by submerging samples into a MeOH bath for 2 min, followed by drying with N_2_. Samples were then submerged in nanopure water for 2 min and dried again with N_2_. One set of three OTCE samples (OTCE-4 MeOH-CV to OTCE-6 MeOH-CV) underwent an additional step of CTAB removal by performing electrochemical cleaning in 100 mM HClO_4_ using a Ag/AgCl electrode (1 M KCl, CH Instruments) as reference and Pt wire as counter in a beaker cell.^57^

After deposition and washing/cleaning of Au NCs, samples were masked with Kapton tape. A 3 mm diameter hole in the center of the Kapton exposed deposited Au NCs (see SI for details), creating a well-defined electroactive area. A Ag/AgCl quasi-reference electrode was made by connecting a Ag wire to the positive terminal and a Pt wire to the negative terminal of a 9 V battery. Both wires were briefly dipped in a saturated KCl solution to form a layer of AgCl on the Ag wire. The wires were then rinsed with nanopure water and dried. A final imaging cell was assembled using imaging spacers to isolate electrodes in a configuration suitable for optical measurements. Briefly, 3 layers of imaging spacers were attached on top of the Kapton tape mask. The Ag/AgCl quasi-reference electrode was then placed diagonally from the top right corner of the imaging spacer, followed by another 3 layers of imaging spacers to secure the electrode. After that, a ring-like Pt wire counter electrode was placed on top of the topmost layer from the top, middle side of the imaging spacer. Lastly, 5 layers of imaging spacers were used to secure the Pt wire. A photograph, schematic and model of the final assembly is shown in SI, Section S4, Figures S2 and S3.

### Electrodissolution of Au NCs

The electrodissolution process of Au NCs was monitored through dark-field microscopy. The schematic of the dark-field microscope setup can be found in SI, Section S4, Figure S4. The Au NC-deposited sample was mounted on an inverted microscope (IX-73, Olympus) with 100x objective (oil immersion, NA 0.6, Olympus). A halogen bulb (12 V, 100W, Microscopical Optical Consulting) was used as the white light source. Light was sent through a dark-field condenser (U-DCD, Olympus) to illuminate the sample with high angle excitation. Low angle scattered light from the Au NCs was collected by the objective and imaged on an EM-CCD camera (Andor iXon Life, Oxford Instruments) at 100 ms acquisition time.

For electrochemical control of the sample, the electrodes were connected to a potentiostat (CH650E, CH Instruments), while the imaging cell was filled with electrolyte solution (125 μL, 0.05 M KClO_4_ in 0.2 M KBr). The appropriate oxidizing potential for Au NCs on each substrate was determined by monitoring the scattering intensity of single Au NCs during LSV (scan rate: 1 mV/s) at every 25 or 50 mV interval. For Au NC electrodissolution experiments, a potential of −0.11 V vs Ag/AgCl was applied for 10s to establish the baseline scattering, followed by an oxidizing overpotential. The duration of the overpotential was 220 s (ITO) and 150 s (OTCE). Data collection at the EM-CCD camera was triggered by the output signal from the potentiostat to synchronize electrochemical and optical data acquisition. The scattering intensity of a single Au NC during the experiment was background-subtracted using a nearby region of interest with an absence of Au NCs on a frame-by-frame basis.

## Results and Discussion

**Figure 2:**
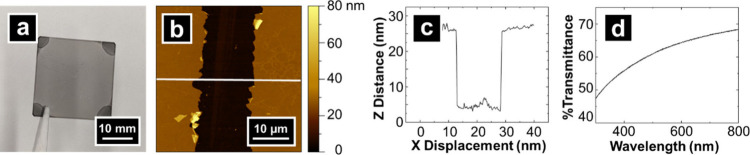
(a) Photograph of an OTCE. (b) AFM image of an OTCE with underlying glass exposed where the white trace represents the line scan in (c). (c) Line scan across OTCE and exposed glass, and (d) plot of percent transmittance in the visible region.

Initially, macroscale characterization of OTCEs was performed and compared to previous studies of similar films. Typical results from topographic and spectroscopic characterization of an OTCE electrode are presented in Figure 2. An OTCE (length = 25.4 mm) is shown in Figure 2a with consistent opacity and clarity to the naked eye. Film thickness and surface roughness were measured by AFM of a deliberately scratched OTCE film (Figure 2b and 2c). From these measurements, a film thickness of 22 nm and an RMS roughness for the film of 0.4 nm were determined (additional AFM data found in SI, Section S5, Figure S5). Film thickness can be widely tuned via experimental parameters.^46−48^ The surface roughness measured here is consistent with previously reported characteristics of PPF-derived substrates,^44,48−50^ and shows considerably lower surface roughness compared to values previously reported for ITO.^36^ Comparison of SEM images of OTCE and ITO (SI, Section S6, Figure S6) shows that on a comparable scale, OTCE is relatively uniform and smooth. Dependent on the type, ITO has grains and crystallites at the submicron scale. Importantly, the uniformity and flatness of OTCEs observed are suitable for correlative electrochemical imaging and electron microscopy of single nanoparticles.

At the film thicknesses prepared here, OTCEs showed 45-70% transmittance (Figure 2d) in the visible region of the spectrum. While this is lower than ITO which has ca. 90% transmittance in the visible region for 0.5 μm film thickness,^58^ previous optoelectrochemical measurements have found that PPF-derived substrates with film thickness similar to OTCEs fabricated in this study provide sufficient transmittance for optoelectrochemical applications,^46−48^ as demonstrated in later sections of this work. Four-point probe measurements of OTCE at thickness ∼22 nm yielded a sheet resistance of 2.84 ± 0.02 kΩ/□ (read as kilo-ohm per square). The conductivity of the OTCE is inversely related to the optical transmission, and thus a balance between film conductivity and transparency, especially for thin films, is key to consider.^44^ Macroscale voltammetric measurements of OTCE were further characterized and showed good electrochemical performance after correcting for sheet resistance (SI, Section S7, Figure S7), in agreement with previous reports.^47,48^

**Figure 3:**
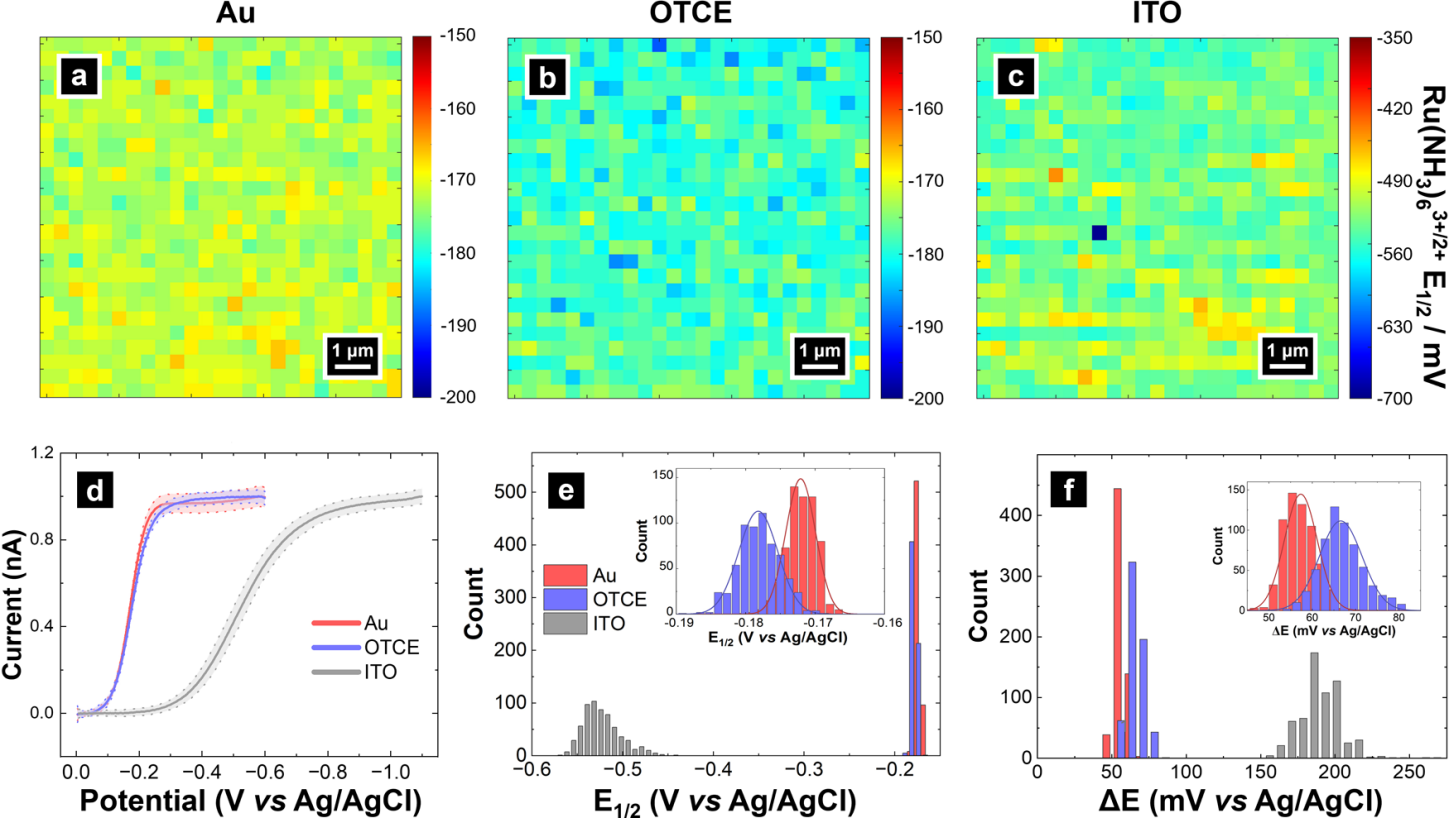
SECCM voltammetric maps of *E*_1/2_ of Ru(NH_3_)_6_^3+^ reduction on (a) Au film, (b) OTCE, and (c) ITO. (d) Average LSV of all pixels collected in the SECCM maps are shown. Histograms of *E*_1/2_ (e) and Δ*E* (f) of the SECCM maps. Solid line is the average response, standard deviation is the dashed line. For data in (d-f), Au (red), OTCE (blue), and ITO (grey) are plotted. Solution: 5 mM Ru(NH_3_)_6_^3+^, 100 mM KCl. Pipette size: 238 nm inner diameter for all scans. Pixel resolution: 400 nm. Scan rate: 1 V/s.

The nanoscale electrochemical response of OTCEs was studied by SECCM voltammetric mapping of ruthenium hexaammine (Ru(NH_3_)_6_^3+^). In addition to OTCE electrodes, comparative SECCM maps of Ru(NH_3_)_6_^3+^ reduction were also collected on Au films, representing a high-quality electrode material, and on ITO-coated coverslips, the predominant choice of transparent electrodes.^36,59−61^ Results of these SECCM measurements are presented in Figure 3. Full SECCM CVs obtained on OTCE are provided in SI, Section S8, Figure S8 and showed sigmoidal responses expected of nanoscale voltammetry with limiting current (*i*_lim_) values of 19.9 ± 0.5 pA. The *i*_lim_ is ca. 10% of that expected at the same sized microdisk electrode, in agreement with previous reports.^54,62,63^ Furthermore, the low magnitude of current passed makes these SECCM measurements effectively immune to ohmic drop.^64^ Taking 5 mV potential shift as a limit of significant impact to voltammetric measurement, 1.76 μA surface current would have to be passed before the sheet resistance of 2.84 kΩ/□ becomes non-negligible and needs to be accounted for.

Forward sweeps of the CVs at each pixel were analyzed as linear sweep voltammograms (LSVs) (Figure 3d) where solid lines represent normalized average LSVs, and dotted traces represent standard deviation. LSVs recorded at OTCE overlap with the Au film with minimal standard deviation. LSVs from ITO showed shifts to more negative (reducing) potentials in the electrochemical response and greater standard deviation in the measured current. The half-wave potential (*E*_1/2_) maps for Au, OTCE, and ITO are presented in Figure 3a-c. Histograms of the *E*_1/2_ values for the three substrates are shown in Figure 3e and highlight the broad distribution of *E*_1/2_ values on ITO as compared to OTCE and Au. This suggests the electrochemical activity on ITO is more spatially heterogeneous than OTCE or Au electrodes. The average *E*_1/2_ measured on OTCE and Au are −0.178 ± 0.003 V and −0.172 ± 0.002 V vs Ag/AgCl (Figure 3a, b, d, and e), agreeing well with the formal potential of ruthenium hexaammine reduction measured on platinum^65^ and carbon substrates.^66,67^ Conversely, ITO showed a shift of *E*_1/2_ to more negative potentials by approximately −354 mV with a distribution of −0.526 ± 0.022 V vs Ag/AgCl (Figure 3c, d, and e), in agreement with trends observed in a previous SECCM study on ITO using ferrocenedimethanol redox probes.^36^ The distribution of quartile potential difference, Δ*E* (E_3/4_ – E_1/4_), estimates^45^ are presented in Figure 3f. OTCE and Au showed Δ*E* = 66.6 ± 4.9 mV and 57.3 ± 3.8 mV, respectively, while ITO showed Δ*E* = 188.0 ± 14.1 mV mirroring the observations for *E*_1/2_. From these results, as per Tomeš criterion of electrochemical reversibility (Δ*E* = 57 mV/n at *T* = 298 K, where n is the number of electrons transferred), OTCE and Au are more favorable for the reversible one-electron transfer Ru(NH_3_)_6_^3+^ reduction process than ITO.^45^ While the Au electrode may have good electron transfer properties from an electrochemical perspective, studies of electrocatalytic reactions such as HER on supported entities (*vide infra*) can be convoluted due to the activity of the underlying Au substrate,^68−71^ making isolation of contributions from nanoparticles of interest difficult.

**Figure 4:**
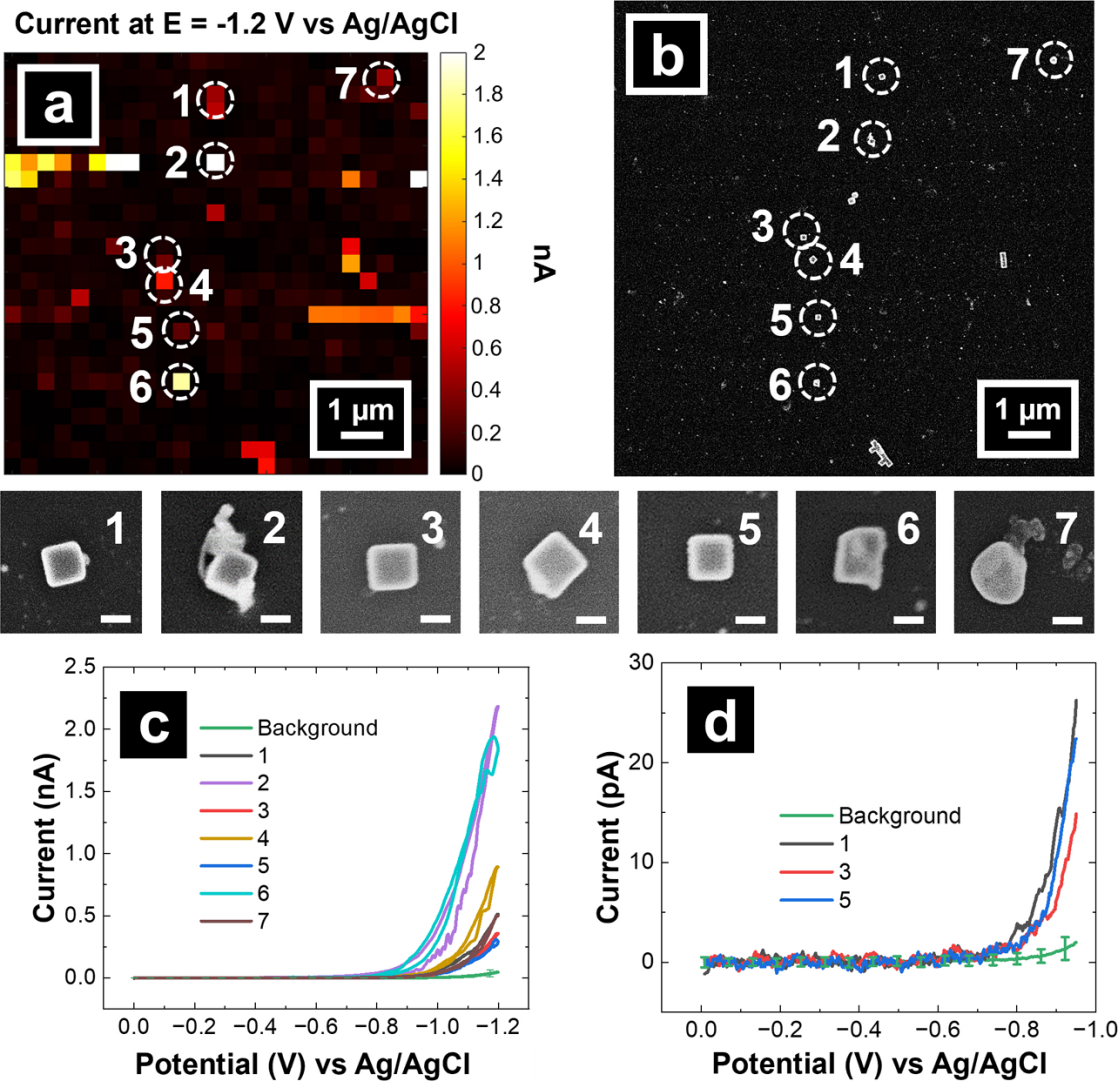
(a) SECCM voltammetric map and (b) correlated SEM image of the SECCM scan area for Au NCs on an OTCE. (c) CVs of HER on seven individual nanoparticles from scan where the number for each trace color relates to the SEM images of the individual nanoparticles directly above the CV plots (scale bar: 100 nm). (d) LSVs of NCs 1, 3, and 5 extrapolated from data shown in (c). Solution: 100 mM HClO_4_. Pipette size: ∼250 nm inner diameter. Pixel resolution: 400 nm. Scan rate: 1 V/s.

To investigate OTCE performance as support electrodes for nanoscale electrochemistry, the HER at Au NCs dispersed on OTCE was studied (Figure 4). The HER (2H^+^ + 2e^–^ ⇌ H_2_) is crucial for producing clean and sustainable energy. Further, the surface sensitivity of the HER provides an ideal reaction for studying surface-function relationships in electrocatalysts.^72,73^ Our previous SECCM studies showed that Au NCs have superior HER activity compared to Au nano-octahedra,^53^ and formed the basis for the present study. SECCM was used to acquire CVs of Au NCs dispersed on an OTCE electrode, using a ∼250 nm probe filled with 50 mM HClO_4_ (see Methods). An SECCM electrochemical map, showing electrocatalytic current at *E* = −1.2 V *vs* Ag/AgCl, is presented in Figure 4a with correlated SEM of the scan area in Figure 4b. Importantly, the locations with NCs in the SEM image correspond to pixels with higher current in the SECCM map. Additional pixels showing higher currents in areas without Au NCs are attributed to meniscus wetting, supported by analysis of the tip current at these locations (SI, Section S8, Figure S9). In a qualitative sense, aside from these pixels, the background current from the OTCE gives a relatively even and low current (SI, Section S8, Figure S10). At *V* = −0.95 V *vs* Ag/AgCl, the magnitude of the HER current on the background OTCE is 2.6 ± 3.9 pA which is within the noise level of the measurement and compares well with observations on glassy carbon at the same potential.^53^ As ITO is known to suffer from instability in acid solutions,^74^ attempts were not made to pursue HER on ITO.

SEM data reveals that NCs 1, 3, and 5 are ideally shaped Au NCs, while nanoparticles 2 and 4 have extraneous materials (residue from ligands and electrolyte solution), and nanoparticles 6 and 7 show imperfect geometries. The voltammetric response of these seven Au NCs as well as the background OTCE are presented in Figure 4c, providing the full potential window scanned from 0 to −1.2 V, while LSVs of the forward sweep of the ideally shaped Au NCs (1, 3, and 5) in a narrowed potential window from 0 to −0.95 V are also presented separately in Figure 4d (correlated electrochemical responses with SEM for Au NC clusters within the SECCM scan are provided in SI, Section S8, Figure S11). Interestingly, the estimated current density for the ideally shaped OTCE-supported Au NCs when normalized to the estimated surface area of the 5 exposed facets of the NC is approximately 25.5 ± 5.6 mA/cm^2^ at *E* = −0.95 V *vs* Ag/AgCl, which is less than the observed values from our prior publication of the HER at Au NCs on glassy carbon at the same potential.^53^ However, this observation may be due to the difference in Au NC size between Choi et al. (78 nm)^53^ and the 130 nm Au NCs used herein since smaller nanoparticles typically have higher electrocatalytic activity.^75^ Overall, these results demonstrate the utility of OTCEs for correlative electrochemical multi-microscopy.

Next, the application of OTCEs to optoelectrochemical studies was examined via electrodissolution studies of Au NCs and compared to ITO substrates. Electrodissolution of Au^0^ in a bromide-rich environment was previously proposed as follows:^76,77^
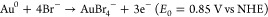

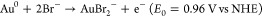


**Figure 5:**
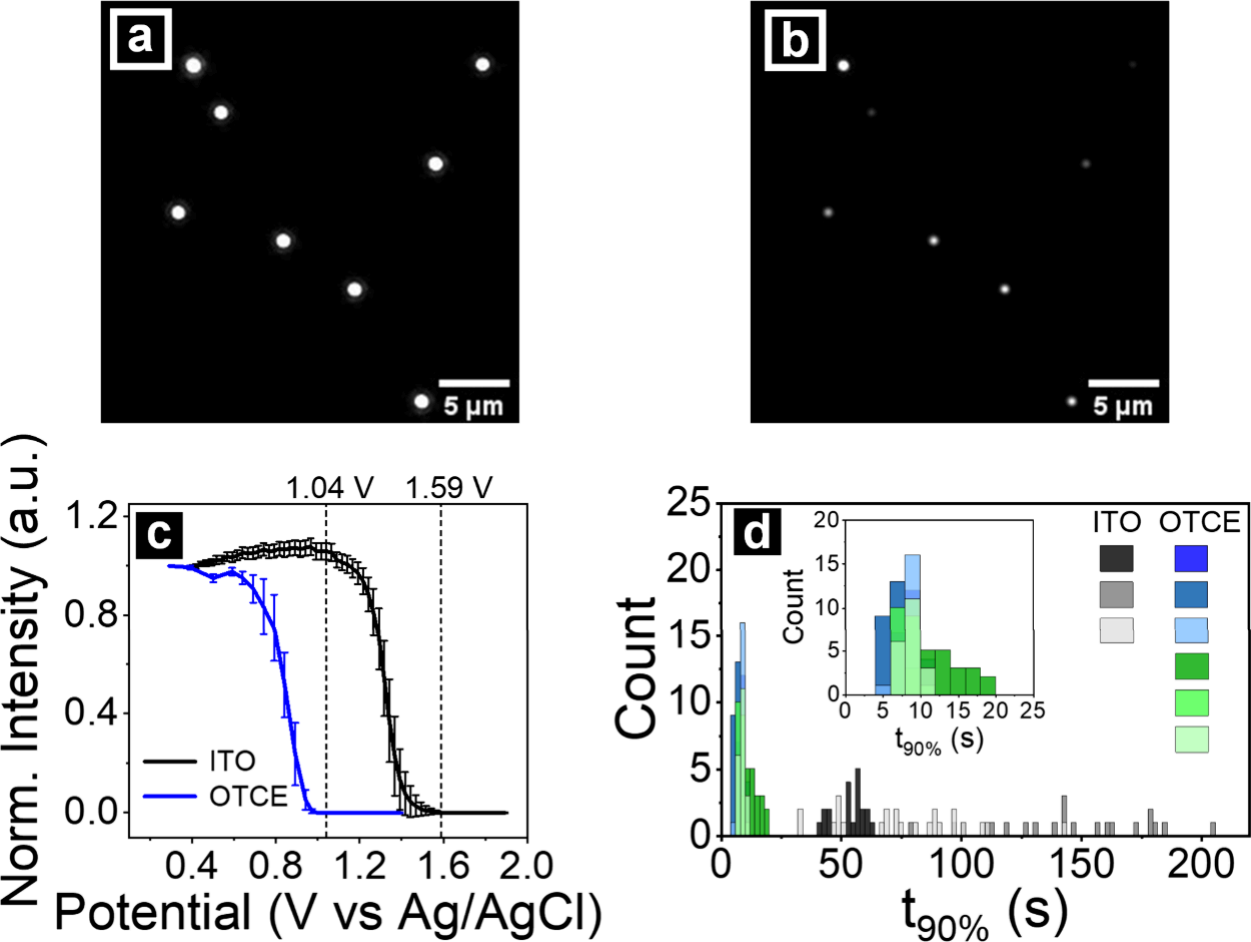
Dark-field image of Au NCs on OTCE in electrodissolution electrolyte (0.05 M KClO_4_ in 0.2 M KBr) at (a) *t* = 0 s (*E* = −0.11 V vs Ag/AgCl) and (b) at*t* = 20 s (10 s after applying *E*_OX, OTC_ = 1.04 V vs Ag/AgCl). (c) Average normalized scattering intensity of single Au NCs on different support electrodes during an LSV experiment at a scan rate of 1 mV/s. The vertical lines represent the overpotentials used for Au NC electrodissolution study on ITO and OTCE in panel (d). (d) Histogram of *t*_90%_ of Au NCs obtained from different support electrodes in the Au NC electrodissolution study. Different shades of black represent 3 different Au NC-deposited ITO samples underwent MeOH-washing process to remove ligands. Shades of blue represent 3 different Au NC-deposited OTCE samples underwent MeOH-washing and shades of green represent 3 different Au NC-deposited OTCE samples underwent MeOH-washing and electrochemical cleaning. Bin size: 2.

Results of electrodissolution studies are presented in Figure 5, with Figure 5a and b showing example dark-field images before and during pulse-potential electrodissolution of Au NCs on OTCE, where the loss in scattering intensity is related to the decrease in NC size as the particle is oxidized. The representative single Au NC scattering intensity time traces during the electrodissolution process can be found in SI, Section S9, Figure S13. Importantly, the scattering intensity of single Au NCs during the potential of −0.11 V observed on ITO and OTCE strongly overlap suggesting that the detection limit of Au NCs on OTCE is very similar to that on ITO (SI, Section S9, Figure S14). These data show that optical imaging of single entities is achieved on OTCEs, despite their lower optical transparency relative to ITO. Figure 5c shows the average normalized nanoparticle scattering intensity as a function of applied potential on both OTCE and ITO substrates and reveals that Au oxidation shifts to more positive potentials on ITO relative to OTCE, similar to the SECCM results in Figure 3 for Ru(NH_3_)_6_^3+^ reduction. The potential shift in the Au NC dissolution waves between the two electrodes could be attributed to the corrosion of the ITO electrode at 0.8–1.1 V vs Ag/AgCl,^78−80^ which could compete with Au dissolution to delay the observed onset of Au NC dissolution, as seen in the optical scattering results. This hypothesis aligns with the known chemical instability of ITO under anodic polarization conditions.^78−80^ The anodic corrosion might be associated with mass loss, as confirmed by quartz crystal microbalance measurements,^78^ and could involve the oxidation of O_2_^-^ from the ITO lattice and the formation of a thin passivating SnO_2_ film.^79,80^ Further, the corresponding linear sweep voltammograms for the optical scattering data in Figure 5c, and current-time traces for constant-potential Au NC dissolution (SI, Section S9, Figure S15) feature anomalous current that supports the occurrence of an additional process on ITO substrate. Voltammograms and i-t traces for ITO show a slight peak (marked with black arrows) that is absent in data acquired on OTCE.

Next, the electrodissolution kinetics of Au NCs on OTCE and ITO electrodes were monitored by first applying a potential of −0.11 V vs Ag/AgCl to measure the pre-oxidation baseline scattering intensity, and then stepping to an oxidizing potential (*E*_OX_) and tracking the change in intensity as the particles dissolved. To ensure complete Au NC electrodissolution in these time-dependent experiments, an additional 50 mV was added to the potential where the scattering intensity of Au NCs disappeared in LSV experiment for each support electrode, such that *E*_OX, OTCE_ = 1.04 V and *E*_OX, ITO_ = 1.59 V. Quantitation of dissolution rates was performed by extracting the time at which the scattering intensity of individual Au NCs dropped to 90% of the initial intensity (*t*_90%_).^39^ Comparison of the electrodissolution performance of Au NCs on ITO vs OTCE is shown in Figure 5d, where all Au NC samples underwent a MeOH-washing process to remove excess CTAB ligand from the sample. One set of OTCE samples underwent an extra electrochemical cleaning step in 100 mM HClO_4_ to promote additional removal of CTAB ligand (green data) as previously described. Attempts to perform this electrochemical cleaning step on ITO samples led to visible deterioration of the ITO film, as has been previously reported for ITO in acidic conditions, further illustrating an advantage of OTCEs for electrochemical studies.^74,81^

While the t_90%_ values for Au NCs on OTCEs are all below 25 s, NCs on ITO showed much longer t_90%_ values that ranged from 25 to >200 s, despite the much larger overpotential being applied to these samples. These observed electrodissolution kinetics time scales on both ITO and OTCE are comparatively longer than electrodissolution/oxidation studies via nanoimpact possible due to the difference in the identity of the nanoparticles^82^ and the shape and size of the chosen nanoparticles.^83−86^ The t_90%_ histograms from the six different OTCEs show strong agreement (Figure 5d, inset), in contrast to the large sample-to-sample heterogeneity observed for ITO (Table S2). Moreover, the varying standard deviations in the t_90%_ histograms on ITO suggest that the substrate has a significant effect on the electrochemical properties of the Au NCs,^39^ while the variation in t_90%_ is much smaller on the OTCEs, suggesting that heterogeneity in the Au NC sample, rather than the support substrates, plays a dominant role in determining electrodissolution time. Results from these experiments highlight improvement in both the inter- and intra-sample heterogeneity of NCs on OTCEs relative to ITO, suggesting that the former substrates are better suited to single entity optoelectrochemistry studies.

An important final aspect of OTCE application to SEE studies is the suitability for widespread adoption, especially for laboratories with different skill sets. Working between multiple laboratories, we found that OTCEs are readily fabricated, with only small procedural adjustments based on equipment and experience required to obtain consistent results. Specifically, conditions optimized at Texas A&M University were implemented at Indiana University using available materials and equipment on-hand as described. Initial experiments using identical parameters to the Texas A&M procedure yielded OTCEs with a transmittance of 34–55% (OTCE-IU1, Table S3) and a sheet resistance of 4.9 ± 0.2 kΩ/□, which is higher than measured at Texas A&M under the same conditions. These results are consistent with incomplete pyrolysis, which was speculated to arise from differences in the effective furnace temperatures between laboratories. An increase in pyrolysis time (from 1 to 3 h and 15 min) was found to generate suitable OTCEs. Four-point probe measurements of six OTCE samples fabricated with these adjusted parameters are reported in Table S3, with the lowest resistivity measured of 2.1 ± 0.1 kΩ/□ and the highest of 2.5 ± 0.2 kΩ/□. Transmittance of these samples ranged from 37-61% (OTCE-IU2 to OTCE-IU7, Table S3).

## Conclusions

In summary, preparation and implementation of OTCEs to address the demand for high-quality transparent support electrodes for SEE has been described. Characterization of films, including by SECCM and optoelectrochemical measurements, clearly demonstrate benefits of OTCEs relative to ITO electrodes. Principally, the electrochemical response was found to be spatially homogenous at the nanoscale compared to ITO, while retaining desirable transparency and conductivity characteristics. The HER of Au NCs on OTCEs was studied with correlative SECCM-SEM, and the electrodissolution of Au NC was studied with optoelectrochemical microscopy, demonstrating utility as a support electrode for multimode single entity nanoelectrochemistry. We hope that this study might motivate future exploration of OTCEs for reliable SEE and correlative nanoelectrochemistry studies.

## Data Availability

Data for this article, including electron microscopy, electrochemical recordings and optical images are available at The Materials Data Facility^87,88^ at 10.18126/t8rn-7h94.

## Supporting Information Available

Additional information and experimental details are available free of charge. Preparation of SECCM Probes; Synthesis of Au Nanocubes (NCs); Electrospray Deposition of Au NCs; Imaging Cell for Optoelectrochemistry Experiments; AFM Image of OTCE Surface; SEM Micrographs of OTCE and ITO Electrodes; Analysis of OTCE with Macroscale CVs; Additional Plots for Nanoscale Characterization with SECCM; Electrodissolution of Au NCs; Interlaboratory Reproducibility Study; Supporting Figures S1–S6 (PDF)
