# Supplementary material for: Optically Transparent Carbon Electrodes for Single Entity Electrochemistry
Source: ACS Electrochem. 2024 Oct 8;1(1):93–102. doi: 10.1021/acselectrochem.4c00048 (PMC11728714; doi:10.1021/acselectrochem.4c00048)
Supplement: Supplementary file 1 — ec4c00048_si_001.pdf [file ec4c00048_si_001.pdf]

Supporting Information for:

Optically Transparent Carbon Electrodes for Single Entity Electrochemistry

Kelly L. Vernon[1], Tipsiri Pungsrisai[2], Oluwasegun J. Wahab[1], Sasha E. Alden[1], Yaxu Zhong[3], Myung-Hoon Choi[1], Ekta Verma[3], Anne K. Bentley[4], Kathleen O. Bailey[1], Sara E. Skrabalak[3], Xingchen Ye[3], Katherine A. Willets[2]*, Lane A. Baker[1]*

Corresponding authors:

Lane A. Baker. Department of Chemistry, Texas A&M University, College Station, Texas 77843

Email: lane.baker@tamu.edu

Katherine A. Willets. Department of Chemistry, Temple University, Philadelphia, 19122

Email: kwillets@temple.edu

[1]Department of Chemistry, Texas A&M University, College Station, Texas 77843, United States

[2]Department of Chemistry, Temple University, Philadelphia, Pennsylvania 19122, United States

[3]Department of Chemistry, Indiana University, Bloomington, Indiana 47405, United States

[4]Department of Chemistry, Lewis & Clark College, Portland, Oregon 97219, United States

Preparation of SECCM Probes S1

Synthesis of Au Nanocubes (NCs) S2

Electrospray Deposition of Au NCs S3

Imaging Cell for Optoelectrochemistry Experiments S4

AFM Image of OTCE Surface S5

SEM Micrographs of OTCE and ITO Electrodes S6

Analysis of OTCE with Macroscale CVs S7

Additional Plots for Nanoscale Characterization with SECCM S8

Electrodissolution of Au NCs S9

Interlaboratory Reproducibility Study S10

Section S1. Preparation of SECCM Probes

Quartz theta capillaries (QT120-90-7.5, Sutter Instruments) were pulled (program: Heat: 690, Filament: 3, Velocity: 35, Delay time: 200, Pull: 150) with a CO_2-laser puller (P-2000, Sutter Instruments) to fabricate dual barrel nanopipette probes. Pipettes were characterized by scanning electron microscopy (SEM, JEOL JSM-7500F), with a typical inner radius of ~220 nm and an outer radius of ~250 nm (**Figure S1**). Both barrels of the pipette were filled with 5 mM $[Ru(NH_3)_6]^{3+}$ in 100 mM KCl electrolyte using a MicroFil syringe (World Precision Instruments). Ag/AgCl wires were back-inserted into each barrel to function as quasi-reference counter electrodes (QRCEs) for SECCM measurements. The QRCEs were calibrated by measuring open-circuit potential against a Ag/AgCl reference electrode (3.5 M KCl, CH Instruments) on a daily basis prior to any electrochemical measurements. Calibration was performed inside a one-compartment electrochemical cell filled with the same solution to be used in the experiment (5 mM $[Ru(NH_3)_6]^{3+}$in 100 mM KCl electrolyte or 100 mM $HClO_4$).

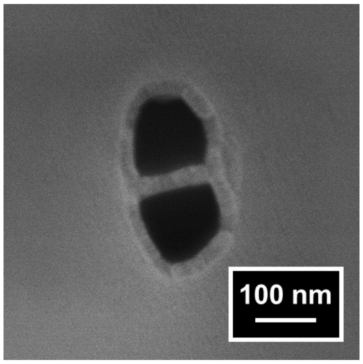

Figure S1. (a) Electron micrograph of pipette tip with ca. 238 nm I.D. across both barrels. Pulling parameters were as follows: HEAT= 690, FIL= 3, VEL= 35, DEL= 200, PULL= 150.

Section S2. Synthesis of Au Nanocubes (Au NCs)

Gold nanocubes (Au NCs) were synthesized using a modified literature method involving iterative cycles of reductive growth and oxidative dissolution reactions.[1] The typical process comprises three main steps: (1) synthesizing gold nanorods (Au NRs), (2) transforming these nanorods into spherical seeds through iterative oxidative dissolution and reductive growth reactions, and (3) seeded growth of Au nanocubes.

(1) *Synthesis of Au NRs*. Au NRs were synthesized using a seed-mediated growth method.[2] In a typical reaction, 125 μL of 10 mM $HAuCl_4$ was mixed thoroughly with 5 mL of 100 mM CTAB in a 20 mL vial. Subsequently, 300 μL of freshly prepared 10 mM $NaBH_4$ solution was quickly injected into the mixture while stirring vigorously. The resulting seed solution was aged at 30 °C for 1 h before use. The growth solution was prepared by sequentially mixing 800 mL of 100 mM CTAB, 40 mL of 10 mM $HAuCl_4$, 7.2 mL of 10 mM $AgNO_3$, and 4.56 mL of 100 mM L-ascorbic acid at 30 °C. Immediately following the addition of L-ascorbic acid, 960 μL of aged seed solution was introduced into the growth solution. The reaction solution was gently stirred for 30 s and left undisturbed at 30 °C for 4 h. The Au NC products were isolated and purified via two rounds of centrifugation at 8000 rpm for 10 min and redispersed into 50 mM CTAB each time. Finally, the optical density (O.D.) of the Au NR solution was adjusted to 2.0 at the longitudinal plasmon peak wavelength by diluting it with a 50 mM CTAB solution (aq).

(2) *Iterative oxidative dissolution and reductive growth of Au NRs into spherical seeds*. Spherical Au seeds were synthesized through iterative reductive growth and oxidative dissolution of Au NRs. Initially, as-synthesized Au NRs were etched by adding a specific volume of 10 mM $HAuCl_4$ to the NR solution (O.D. = 2) to achieve a final Au^{3+}

concentration of 75 μM. The solution was then kept at 40 °C with stirring (ca. 200 rpm) for 4 h. The products were purified using two rounds of centrifugation at 8200 rpm for 20 min and followed by redispersion in 100 mM cetylpyridinium chloride (CPC) after each round. Next, reductive growth of etched Au NRs into concave rhombic dodecahedra (CRD) and subsequent oxidative dissolution were conducted. To grow Au CRD, 10 mL of 10 mM CPC, 175 μL of 10 mM $HAuCl_4$, and 2.25 mL of 100 mM L-ascorbic acid were mixed sequentially, after which 3 mL of the etched Au NR solution (O.D. = 1.0) was added to this mixture. The solution was gently stirred for 30 s and left undisturbed at 40 °C for 15 min. The resultant Au CRDs were purified through two rounds of centrifugation at 8000 rpm for 10 min followed by redispersion in 50 mM CTAB after each centrifugation step. Subsequent etching of the Au CRDs (O.D. = 1.0) was performed by adding a specific volume of 10 mM $HAuCl_4$ to reach a final Au^{3+} concentration of 60 μM. The reaction solution was kept at 40 °C under stirring for 4 h, after which the resultant spherical seeds were isolated through two rounds of centrifugation at 8200 rpm for 10 min. The spherical seeds were finally dispersed in 100 mM CPC to reach O.D. of 1.0 at 524 nm.

(3) *Synthesis of Au NCs*. Typically, 0.5 mL of the spherical seed solution (O.D. = 1.0 at 524 nm) was added to a mixture of 25 mL of 100 mM CPC, 2.5 mL of 100 mM KBr, 1.5 mL of 10 mM $HAuCl_4$, and 2.25 mL of 100 mM L-ascorbic acid. The solution was gently stirred for 30 s and then left undisturbed at 30 °C for 1 h. The resulting Au NCs were purified by centrifugation at 3000 rpm for 3 min, followed by redispersion in 50 mM CTAB solution. The Au NCs were further purified with another round of centrifugation at 2000 rpm for 3 min. Finally, the Au NCs were dispersed in 2 mL of 20 mM CTAB for future use.

Section S3. Electrospray Deposition of Au NCs

Electrospray deposition was employed to prepare samples for SECCM measurements which limits particle clusters or aggregation as previously demonstrated.[3] Borosilicate theta capillaries (BT-150-10, Sutter Instruments) were pulled (program: Heat: 700, Pull: 0, Velocity: 16, Time: 35) on a filament puller (P-97, Sutter Instruments). The resultant borosilicate micropipette had an inner diameter of 1.2 μm. One barrel was filled with colloidal Au NCs solution (O.D.= 8 in 20 mM CTAB) in 1:10 v/v dilution with Milli-Q Water and the other barrel with 2.5 mM KCl electrolyte solution. A Pt wire was back-inserted into the barrel with the Au NC solution and a high voltage was applied to the Pt wire. The electrospray current was collected at the OTCE substrate and monitored with a picoammeter (414S, Keithley Instruments). For accurate positional control of the pipette emitter over the OTCE substrate, a robotic arm was used (DOBOT Magician, In-Position Technologies). A digital microscope (YPC-X03, Inskam) was used to determine the distance between the pipette emitter tip and the OTCE substrate to ensure the distance was constant throughout the electrospray process. A humidity-controller chamber sealed the electrospray setup to keep the relative humidity between 15-20%. A constant current of ca. 8 nA was employed during electrospray for 20 min. The OTCE electrodes loaded with Au nanocrystals underwent methanol rinsing then electrochemical cleaning as detailed in previous work to remove CTAB ligands.[4]

Section S4. Imaging Cell for Optoelectrochemistry Experiments

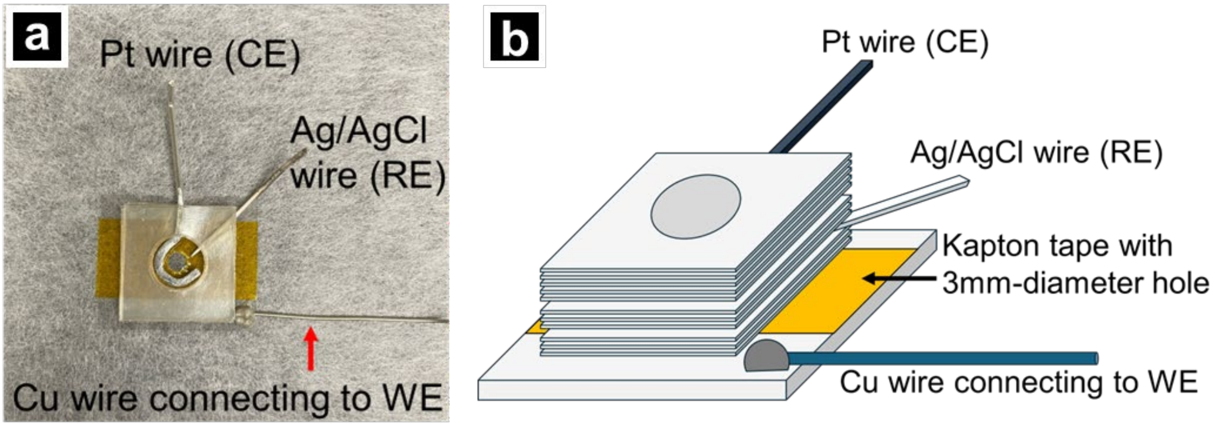

Figure S2. (a) photograph of the final imaging cell. (b) schematic of the final imaging cell.

This electrochemical cell design was adopted in an attempt to minimize changes in current density due to electrode placement. Finite element simulations of a simulated geometry (model report included) showed more homogeneous electric fields relative to other configurations.

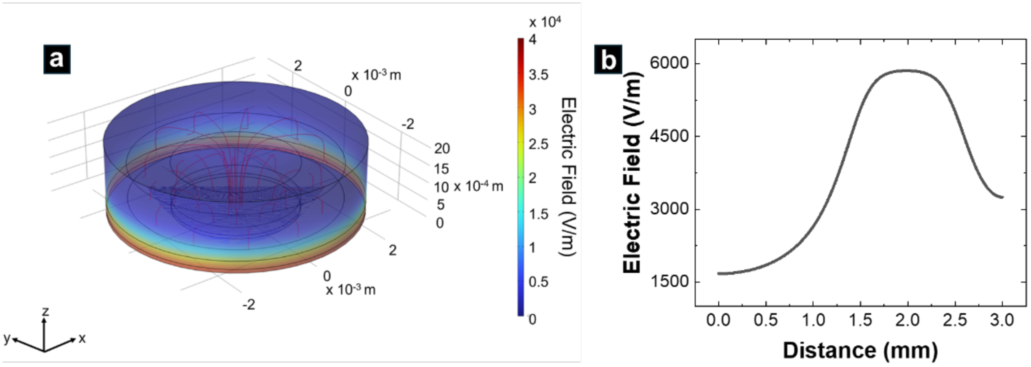

Figure S3. **COMSOL model** (a) 2D axis symmetric finite element simulation of the electric field within the dissolution cell. Field lines are shown in red; field strength is indicated by color scale. Potential applied to working electrode, OTCE, was 1V. Potassium chloride was used as a model electrolyte at 100mM concentration. (b) Strength of the electric field across the working electrode surface, extracted from 2D axis symmetric model, as a function of distance from the axis. Field increases as it approaches the counter electrode, shown at 1.5 - 2.5 mm distance from the axis.

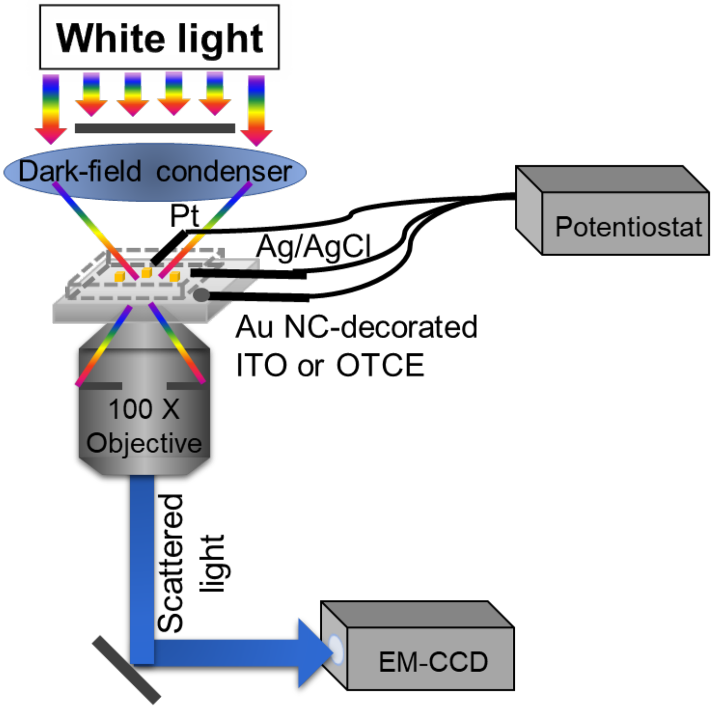

Figure S4. Schematic of dark-field microscope setup for Au NC electrodissolution experiments. The Au NCs on a supporting electrode were illuminated with the high-angled light that passed through a dark-field condenser. The scattered light from Au NCs were collected through a low NA objective and imaged on an electron-multiplying CCD. Details on the final imaging cell can be found in **Figure S2**.

Section S5. AFM Image of OTCE Surface

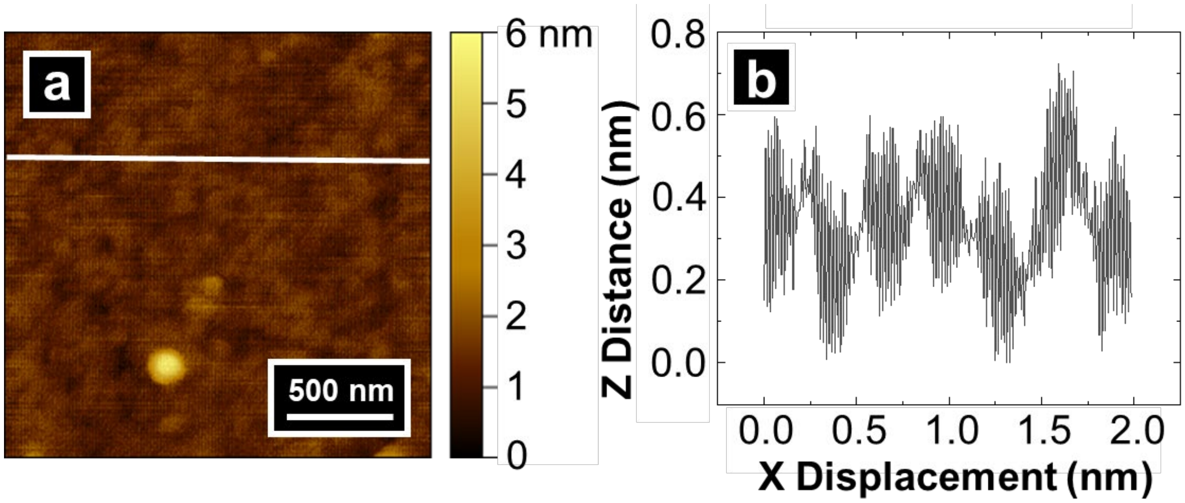

Figure S5. (a) AFM image of OTCE surface. White trace in (a) represents the median line scan for (b). (b) Average line profile of 100 line scans from (a).

Section S6. SEM Micrographs of OTCE and ITO Electrodes

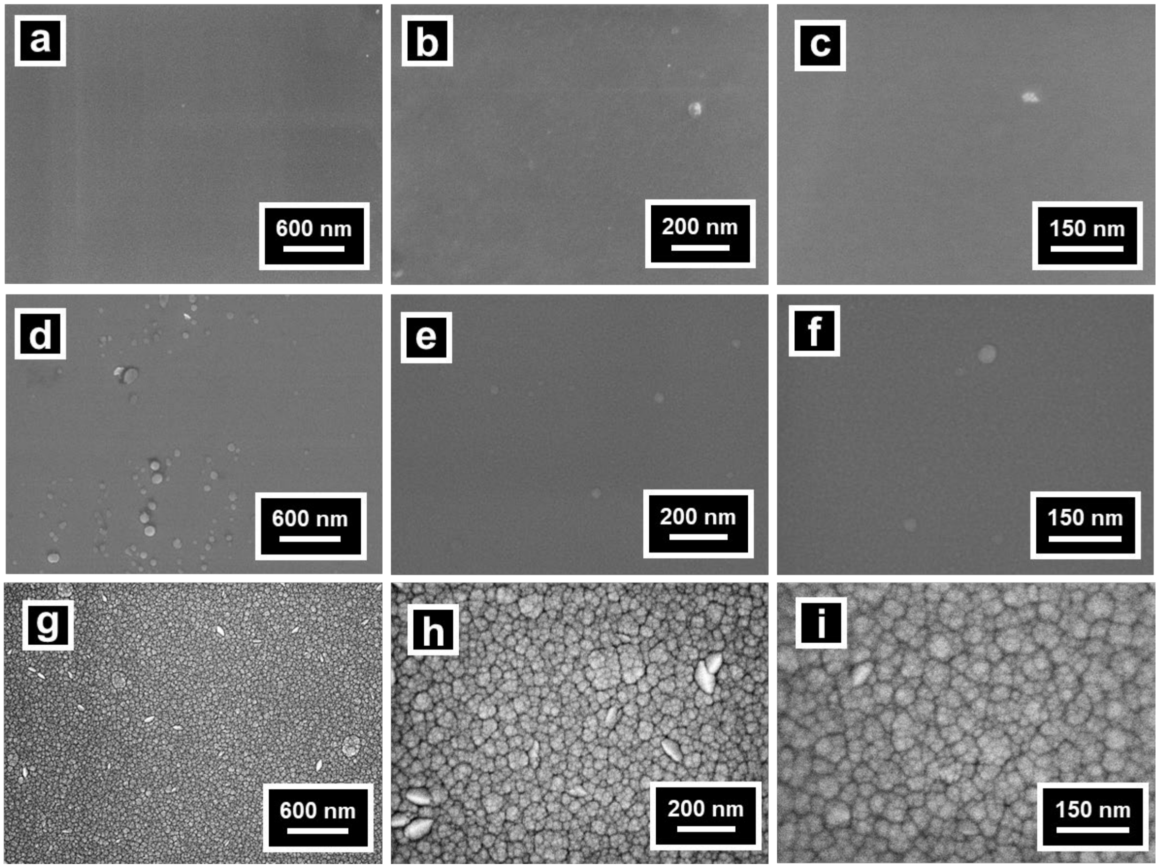

Figure S6. Electron micrographs of bare (a-c) OTCE, (d-f) ITO (70-100 Ω/□), and (g-i) ITO (8-12 Ω/□).

Section S7. Analysis of OTCE with Macroscale CVs

Sheet resistance measurements were taken with a four-point probe (Ossila) under parameters: Sample Geometry = Rectangular, Long Side (mm) = 25.4, Short Side (mm) = 25.4, Readings to Save = 25.

Equations S1-4 are used for iR compensation for macroscale measurements of OTCE:

$$R = R_s \times \frac{L}{w} \tag{S1}$$

$$(E_{pc})_{corr} = E_{pc} - (i_{pc})R \tag{S2}$$

$$(E_{pa})_{corr} = E_{pa} - (i_{pa})R \tag{S3}$$

$$(\Delta E_p)_{corr} = |(E_{pc})_{corr} - (E_{pa})_{corr}| \tag{S4}$$

Where R is bulk resistance, R_s is sheet resistance, L is length of electrode surface, w is width of electrode surface, $(E_{pc})_{corr}$ is the corrected cathodic peak potential, and E_{pc} and i_{pc} are cathodic peak potential and current. $(E_{pa})_{corr}$ is the corrected anodic peak potential, E_{pa} and i_{pa} are anodic peak potential and current, and $(\Delta E_p)_{corr}$ is the corrected change in potential. Bulk resistance is equal to sheet resistance for square electrode areas used.

Table S1. Correction for ΔE_p due to iR where R_s = 2251 Ω/□ for scan rates across concentrations of ruthenium(III) hexaammine $[Ru(NH_3)_6]^{3+}$.

Scan Rate (mV/s)	0.125 mM $[Ru(NH_3)_6]^{3+}$ in 100 mM KCl		0.25 mM $[Ru(NH_3)_6]^{3+}$ in 100 mM KCl		0.5 mM $[Ru(NH_3)_6]^{3+}$ in 100 mM KCl		1 mM $[Ru(NH_3)_6]^{3+}$ in 100 mM KCl	
	ΔE_p (mV)	$(\Delta E_p)_{corr}$ (mV)	ΔE_p (mV)	$(\Delta E_p)_{corr}$ (mV)	ΔE_p (mV)	$(\Delta E_p)_{corr}$ (mV)	ΔE_p (mV)	$(\Delta E_p)_{corr}$ (mV)
10	76	73	80	69	93	71	109	69
25	71	66	80	64	93	61	126	67
50	71	64	84	62	107	63	146	67
100	74	65	95	65	120	61	178	76

CVs of $[Ru(NH_3)_6]^{3+}$ on OTCEs were taken in a three-electrode set-up. For bulk electrochemical characterization, a Kapton tape mask was applied to OTCE to expose a 4 mm circle to define the WE area. Electrical contact was made to the OTCE via copper tape on the electrode sealed under Kapton tape. The CE was Pt mesh and RE was a Ag/AgCl (3.5 M KCl) electrode. The concentration of $[Ru(NH_3)_6]^{3+}$ was varied (0.125 M, 0.25 M and 0.5 M) across a range of scan rates (5 mV/s, 10 mV/s, 25 mV/s, 50 mV/s 100 mV/s, and 1 V/s). Cathodic peak current versus the square root of the scan rate was extrapolated from the CVs and revealed linear trends across concentrations. For data taken here, CVs were measured without electronic compensation and were only corrected for sheet resistance as described above.

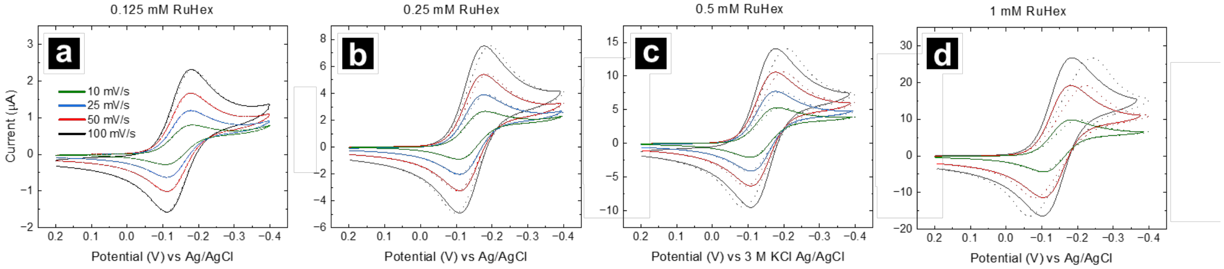

Figure S7. CVs of reduction of $[Ru(NH_3)_6]^{3+}$ on OTCE where the concentration of $[Ru(NH_3)_6]^{3+}$ is (a) 0.125 mM, (b) 0.25 mM, (c) 0.5 mM, and (d) 1 mM. Dotted lines are the CVs for each scan rate without iR compensation. Solid lines are the CVs after iR compensation.

Section S8. Additional Plots for Nanoscale Characterization with SECCM

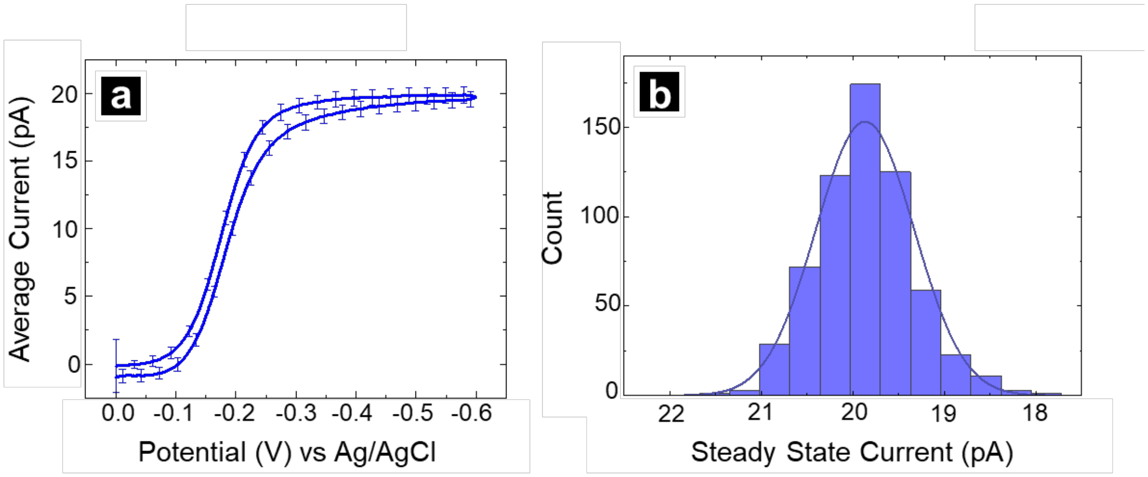

Figure S8. Additional analysis of SECCM data presented in Figure 3b. Averaged SECCM CV from OTCE current map, error bars represent standard deviation. N = 625.

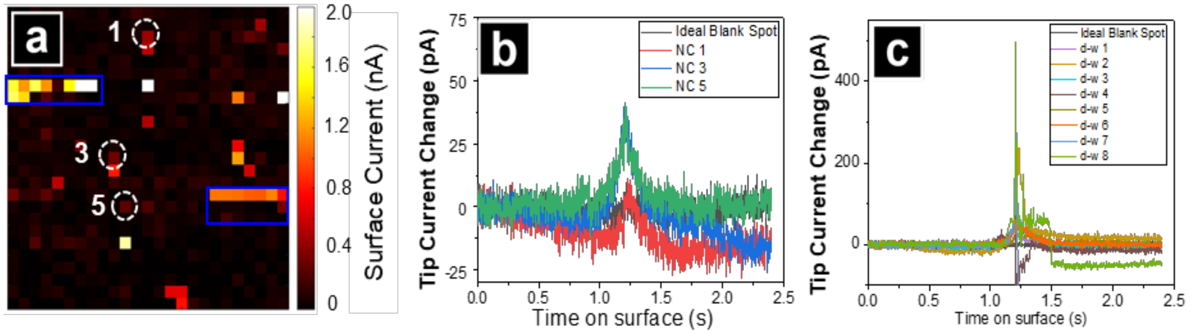

Figure S9. (a) SECCM map of Au NCs on OTCE as presented in Figure 4 of the main text. Pixels corresponding to nanocrystals 1, 3, and 5 are highlighted as in the main text. Blue boxes to the top left and bottom right mark regions of high surface current from wetting. (b) Plots comparing tip current traces for location on nanocrystals to a location on blank OTCE with an ideal wetting. (c) Plots comparing tip current traces for location on blank OTCE with larger wetting to a location on blank OTCE with an ideal wetting. Irregularities and high current in (c) compared to (b) are indicative of meniscus instability or wetting. Furthermore, the pattern of the wetted pixels with high current aligned with the direction of probe travel and suggests that the meniscus merged with the footprint of adjacent landing sites. The two streaks observed are 8 and 10 pixels long, providing a rough estimate of the maximum wetting. Aside from these wetted pixels, there is no indication of merged meniscus footprints in the remaining part of the scan which confirms that the prevailing meniscus wetting is less than the probe hopping distance (400 nm) and therefore comparable to the probe size of approximately 200 nm.

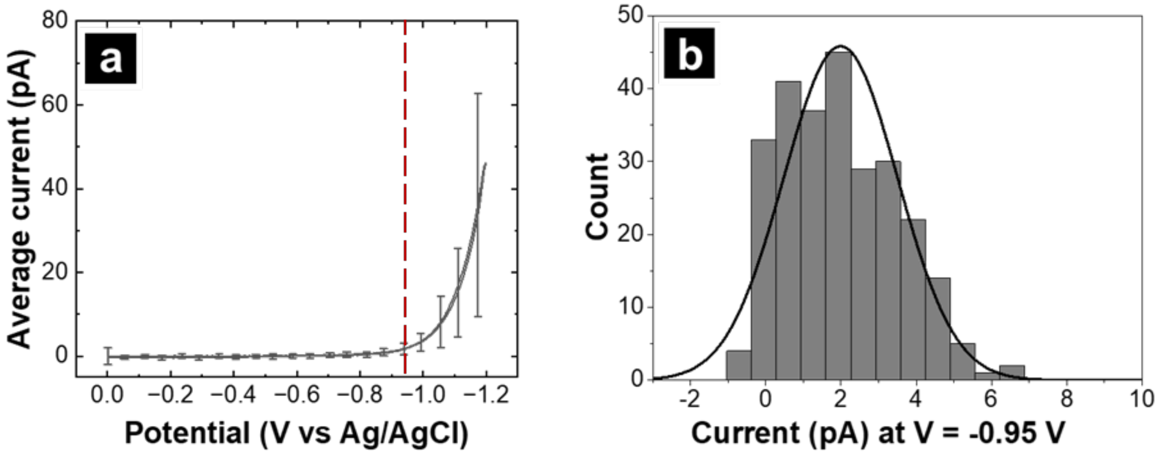

Figure S10. Additional analysis of SECCM data presented in Figure 4. (a) Averaged SECCM CV from 255 background pixels in current map, error bars represent standard deviation. The red dashed line indicates the potential at which the data in (b) is extracted. (b) Histogram of background HER current on OTCE at V = -0.95 V *vs* Ag/AgCl.

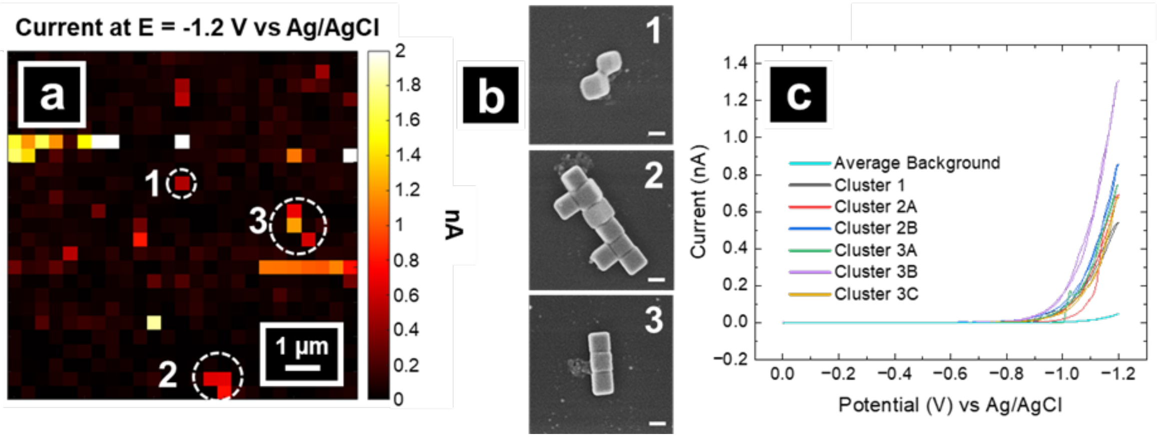

Figure S11. (a) SECCM voltammetric map of the SECCM scan area. (b) Electron micrographs of 3 clustered Au NCs, 8 clustered Au NCs, and 3 clustered Au NCs (scale bar: 100 nm). (c) CVs of HER on three clusters from scan where the number for each trace color relates to the SEM images of the clusters shown in (b) and the letters relate to the pixels that show activity in the correlated location.

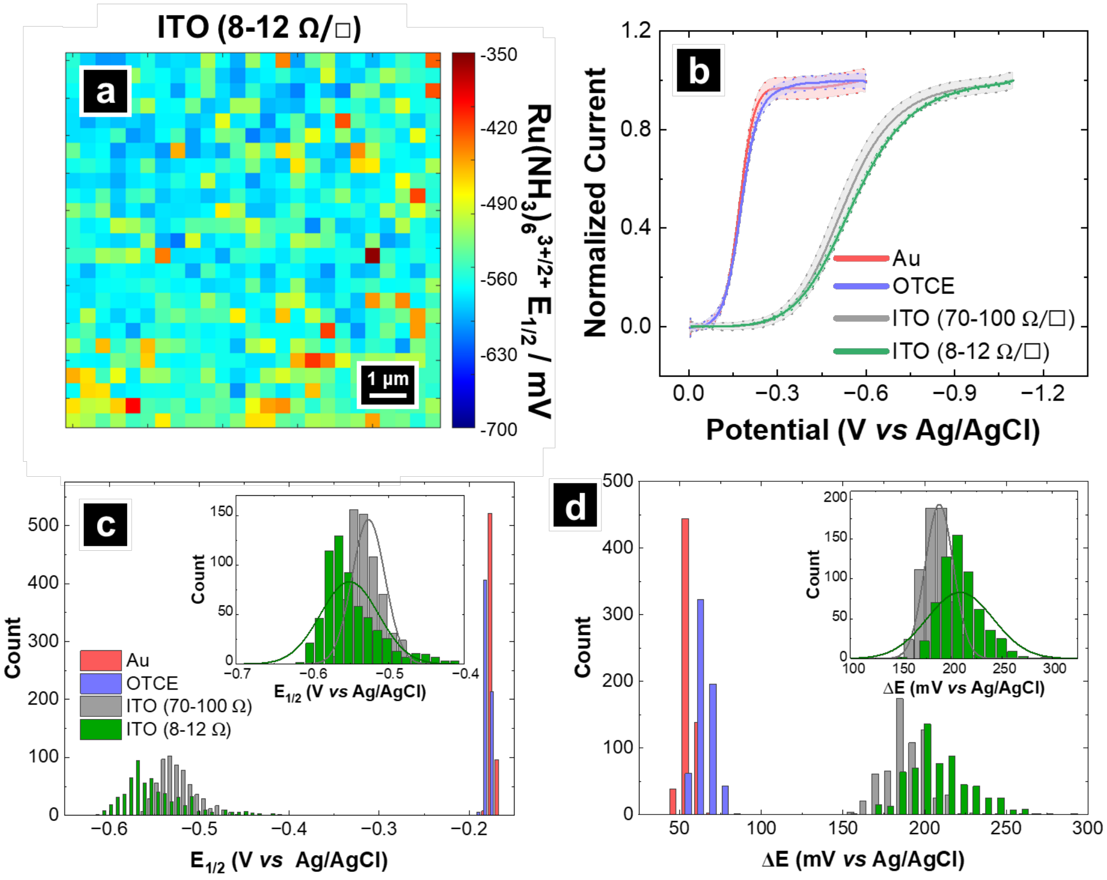

Figure S12. (a) SECCM voltammetric map of $E_{1/2}$ of $Ru(NH_3)_6$ reduction on OTCE. (b) Average LSV of all pixels collected in the SECCM maps from **Figure 3** and **Figure S10a** are shown. Histograms of (c) $E_{1/2}$ and (d) ΔE of the SECCM maps. Solid line is the average response, standard deviation is the dashed line. For data in (d-f), red represents Au, blue represents OTCE, and grey represents ITO. Solution: 5 mM $Ru(NH_3)6^{3+}$ in 100 mM KCl. Pipette size: 238 nm inner diameter for all scans. Scan rate: 1 V/s.

Section S9. Electrodissolution of Au NCs

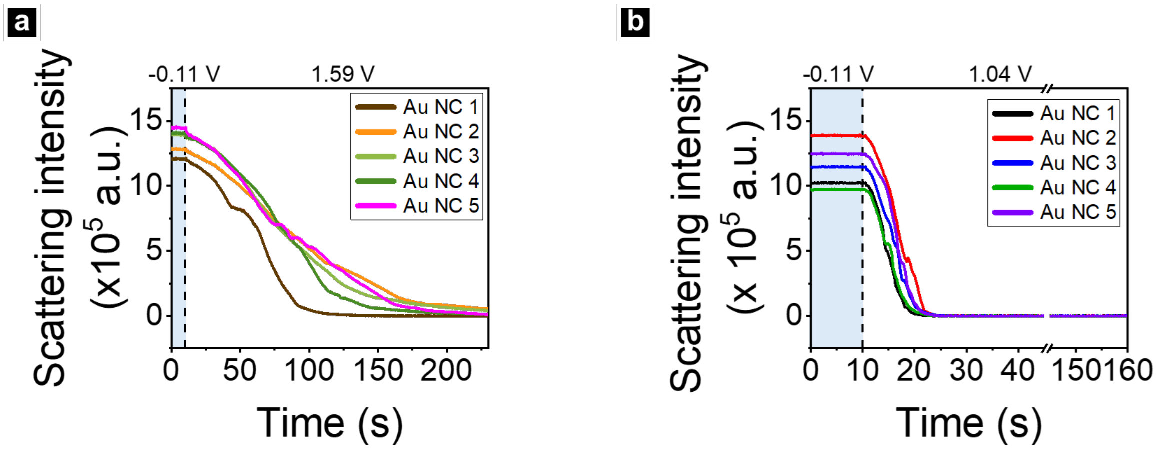

Figure S13. Scattering intensity time traces of representative single Au NCs during the electrodissolution experiment on (a) ITO-2 MeOH-washed and (b) OTCE-1 MeOH-washed. The blue area indicates the time the applied potential was set to -0.11V to establish the baseline scattering intensity of individual Au NCs. The dashed-vertical line represents the time the applied potential switched from -0.11 V (a) to 1.59 V vs Ag/AgCl on ITO and (b) to 1.04 V vs Ag/AgCl on OTCE.

Table S2. Substrate-dependent electrodissolution kinetics of Au NCs

Sample	Average $t_{90\%}$ (s)	Standard deviation (s)	N
ITO-1 MeOH-washed	76.3	26.8	24
ITO-2 MeOH-washed	144.4	32.8	20
ITO-3 MeOH-washed	53.4	6.6	20
OTCE-1 MeOH-washed	8.4	1.1	21
OTCE-2 MeOH-washed	6.1	0.6	22
OTCE-3 MeOH-washed	9.2	1.2	21
OTCE-4 MeOH-CV	13.4	2.9	21
OTCE-5 MeOH-CV	8.1	0.9	20
OTCE-6 MeOH-CV	8.6	1.1	20

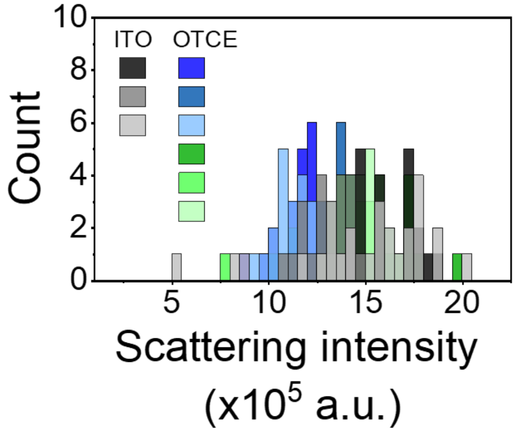

Figure S14. Histogram of average scattering intensity of single Au NCs on different supporting electrodes over 10 s of applying the potential of -0.11 V vs Ag/AgCl for baseline scattering intensity. Different shades of black represent average scattering intensity of single Au NCs deposited on 3 different ITO samples underwent MeOH-washing process to remove ligands. Shades of blue represent average scattering intensity of single Au NCs deposited on 3 different OTCE samples underwent MeOH-washing and shades of green represent average scattering intensity of single Au NCs deposited on 3 different OTCE samples underwent MeOH-washing and electrochemical cleaning.

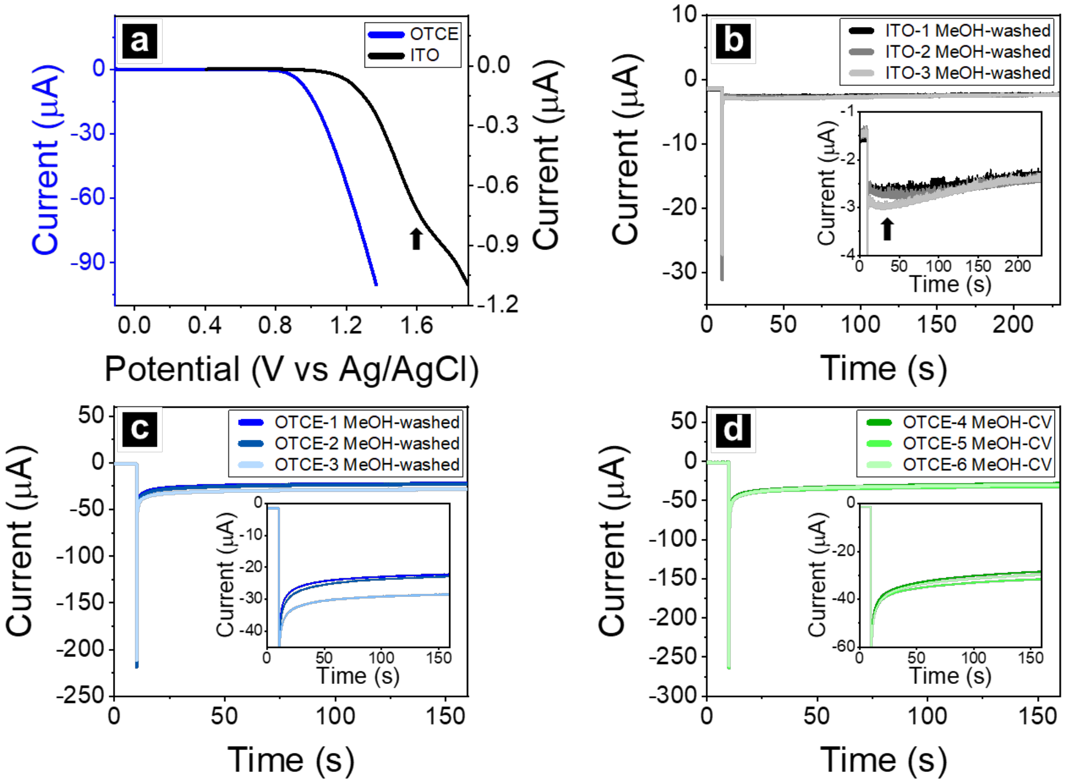

Figure S15. Current-time traces obtained in 0.05 M $KClO_4$ in 0.2 M KBr electrolyte solution during (a) LSV experiments on ITO and OTCE and (b)-(d) electrodissolution experiments on different supporting electrodes. (b) ITO underwent MeOH-washing to remove ligands (c) OTCE-1 to -3 underwent MeOH-washing process and (d) OTCE-4 to -6 underwent MeOH-washing process and electrochemical cleaning. The black arrows in panel a and b inlet indicate additional process on ITO during the electrodissolution experiment.

Section S10. Interlaboratory Reproducibility Study

Interlaboratory Reproducibility Study. OTCE was fabricated as described earlier but with the following variations. *Materials:* S1813 photoresist (Kayaku Advanced Materials Inc S1813 Positive Photoresist, Fischer Scientific), *Equipment:* The photoresist solution was spin-coated on coverslips using Headway PWM50-DY-R790 Spinner. The tube furnace used for pyrolysis was OTF-1200X, MTI Corporation, CA. The optimized pyrolysis conditions were found to be 3 hours and 15 minutes. The thickness of the resulting

OTCEs was characterized by atomic force microscopy (AFM, Asylum Research MFP-3D) and transmittance (Varian CARY 100 UV-visible Spectrophotometer).

Table S3. Characterization of OTCEs from interlaboratory reproducibility study.

Sample	% Transmittance (300-800 nm)	Sheet Resistance (kΩ/□)
OTCE-IU1	35-54	4.87 ± 0.21
OTCE-IU2	40-61	2.33 ± 0.06
OTCE-IU3	41-61	2.41 ± 0.29
OTCE-IU4	39-60	2.28 ± 0.12
OTCE-IU5	39-59	2.12 ± 0.15
OTCE-IU6	37-58	2.07 ± 0.14
OTCE-IU7	37-57	2.47 ± 0.19

REFERENCES

(1) O'Brien, M. N.; Jones, M. R.; Brown, K. A.; Mirkin, C. A. Universal Noble Metal Nanoparticle Seeds Realized Through Iterative Reductive Growth and Oxidative Dissolution Reactions. *J. Am. Chem. Soc.* **2014**, *136* (21), 7603–7606. https://doi.org/10.1021/ja503509k.

(2) Nikoobakht, B.; El-Sayed, and M. A. Preparation and Growth Mechanism of Gold Nanorods (NRs) Using Seed-Mediated Growth Method. *Chem. Mater.* **2003**, *15*, 1957–1962.

(3) Jagdale, G. S.; Choi, M.-H.; Siepser, N. P.; Jeong, S.; Wang, Y.; Skalla, R. X.; Huang, K.; Ye, X.; Baker, L. A. Electrospray Deposition for Single Nanoparticle Studies. *Anal. Methods* **2021**, *13* (36), 4105–4113. https://doi.org/10.1039/D1AY01295A.

(4) Jeong, S.; Choi, M.-H.; Jagdale, G. S.; Zhong, Y.; Siepser, N. P.; Wang, Y.; Zhan, X.; Baker, L. A.; Ye, X. Unraveling the Structural Sensitivity of CO2 Electroreduction at Facet-Defined Nanocrystals via Correlative Single-Entity and Macroelectrode Measurements. *J. Am. Chem. Soc.* **2022**, *144* (28), 12673–12680. https://doi.org/10.1021/jacs.2c02001.
